# Synergistic Effects of Chemotherapy and Phototherapy on Ovarian Cancer Using Follicle-Stimulating Hormone Receptor-Mediated Liposomes Co-Loaded with SN38 and IR820

**DOI:** 10.3390/pharmaceutics16040490

**Published:** 2024-04-02

**Authors:** Lina Pian, Bowen Zeng, Nuoya Wang, Shuangqing Wang, Hao Wu, Hongshuang Wan, Liqing Chen, Wei Huang, Zhonggao Gao, Dan Jin, Mingji Jin

**Affiliations:** 1 Immunology Biology Key Laboratory, Yanbian University, Yanji 133000, China; pianlina2024@yeah.net; 2 Department of Gynecology, Yanbian University Hospital, Yanji 133000, China; 3 State Key Laboratory of Bioactive Substance and Function of Natural Medicines, Institute of Materia Medica, Chinese Academy of Medical Sciences and Peking Union Medical College, Beijing 100050, China; zengbowen1205@yeah.net (B.Z.); wangnuoya@zgyxkxyywyjs.wecom.work (N.W.); haowu@jlmu.edu.cn (H.W.); 2019001080@ybu.edu.cn (H.W.); chenliqing@imm.ac.cn (L.C.); huangwei@imm.ac.cn (W.H.); jinmingji@imm.ac.cn (M.J.); 4 Beijing Key Laboratory of Drug Delivery Technology and Novel Formulations, Institute of Materia Medica, Chinese Academy of Medical Sciences and Peking Union Medical College, Beijing 100050, China

**Keywords:** FSHβ (33–53) peptide, ovarian cancer, targeting therapy, PDT, SN38

## Abstract

We have developed an ovarian cancer-targeted drug delivery system based on a follicle-stimulating hormone receptor (FSHR) peptide. The lipophilic chemotherapeutic drug SN38 and the photosensitizer IR820 were loaded into the phospholipid bilayer of liposomes. The combination of chemotherapy and phototherapy has become a promising strategy to improve the therapeutic effect of chemotherapy drugs on solid tumors. IR820 can be used for photodynamic therapy (PDT), effectively converting near-infrared light (NIR) into heat and producing reactive oxygen species (ROS), causing damage to intracellular components and leading to cell death. In addition, PDT generates heat in near-infrared, thereby enhancing the sensitivity of tumors to chemotherapy drugs. FSH liposomes loaded with SN38 and IR820 (SN38/IR820-Lipo@FSH) were prepared using thin-film hydration-sonication. FSH peptide binding was analyzed using 1H NMR spectrum and Maldi-Tof. The average size and zeta potential of SN38/IR820-Lipo@FSH were 105.1 ± 1.15 nm (PDI: 0.204 ± 0.03) and −27.8 ± 0.42 mV, respectively. The encapsulation efficiency of SN38 and IR820 in SN38/IR820-Lipo@FSH liposomes were 90.2% and 91.5%, respectively, and their release was slow in vitro. FSH significantly increased the uptake of liposomes, inhibited cell proliferation, and induced apoptosis in A2780 cells. Moreover, SN38/IR820-Lipo@FSH exhibited better tumor-targeting ability and anti-ovarian cancer activity in vivo when compared with non-targeted SN38/IR820-Lipo. The combination of chemotherapy and photodynamic treatment based on an FSH peptide-targeted delivery system may be an effective approach to treating ovarian cancer.

## 1. Introduction

Ovarian cancer, known as the “silent killer”, has the highest mortality rate of all gynecological tumors [1], and over 90% of ovarian cancers are epithelial carcinomas [2]. More than 60% of ovarian cancers are diagnosed in the late stages, due to the lack of clinical manifestations in the early stages [3,4]. Ovarian cancer originates from the ovarian surface epithelial cells or the fallopian tube secretory epithelial cells and malignant cells can spread directly to the peritoneum, resulting in metastasis and poor treatment outcomes [5]. Improvements in ovarian cancer treatments are urgently needed. Current research is focused on accurately targeting cancer cells and enhancing the efficacy of therapeutic drugs while reducing side effects. Chemotherapy remains the most important treatment modality for ovarian cancer. At present, the first-line chemotherapy drug for ovarian cancer is platinum plus paclitaxel, but due to the resistance and high recurrence rate of platinum plus paclitaxel, many patients cannot receive effective treatment [6]. Perhaps choosing different types of chemotherapy and chemotherapy administration methods can improve treatment efficacy and reduce patient recurrence and mortality rates [7]. However, many chemotherapy drugs have poor molecular water solubility, short circulating half-life, and severe cytotoxicity to healthy organs, which limits their clinical application.

SN38 (7-ethyl-10-hydroxythectothecin) is an active metabolite of irinotecan (CPT-11) and a derivative of camptothecin obtained through chemical structure modification. It has a strong anti-tumor effect and high anti-tumor activity [8]. The biological activity of SN38 is 100–1000 times that of irinotecan for some tumor cell lines [9]. However, due to the relatively high hydrophobicity and instability at physiological pH, clinical use of SN38 is limited currently [10]. Recently, a variety of drug delivery strategies have been developed to overcome the shortcomings of SN38. Many attempts have been made to develop intravenous or oral preparations of SN38 with better anti-tumor effects. Liposomes are small spherical vesicles derived from naturally occurring non-toxic phospholipids and are biodegradable nanosystems, which have been extensively utilized as drug delivery systems. Onivyde (Irinotecan liposome injection), a pioneer in the development of liposomes loaded with camptothecin and its derivatives, is used to treat patients with tumors. Although SN38-based chemotherapy improved the efficacy of ovarian cancer treatment, drug resistance develops in cancer cells and serious side effects occur. To overcome the inherent limitations of single chemotherapy drugs, chemotherapy has been combined with phototherapy induced by photosensitizers as a practical clinical cancer treatment platform.

Photodynamic therapy (PDT) combined with chemotherapy is an emerging field of chemical phototherapy that has emerged as a promising strategy to improve the efficacy of chemotherapy drugs in the treatment of cancer cells [11]. PDT can efficiently convert near-infrared light (NIR) into heat and produce reactive oxygen species (ROS), causing damage to intracellular components and leading to cell death. In addition, PDT generates heat in the near-infrared, thereby enhancing the efficiency of chemotherapy drug uptake by increasing the permeability of cancer cell membranes [11]. Indocyanine green (IR820) is a clinical infrared fluorescent dye that efficiently absorbs laser light for photothermal and PDT. IR820 is non-toxic until exposed to laser irradiation. The limitations of IR820 include rapid clearance (blood half-life of 2–4 min) and low cellular uptake. These limitations restrict the diagnostic and treatment applications of IR820. Enhancing the intracellular release of chemotherapeutic drugs during the PDT process is essential to amplifying the synergistic therapeutic effects of chemo-photodynamic therapy.

Due to the different specific markers expressed on the surface of tumor cells and normal cells, peptides and antibody ligands that can bind to tumor specific markers are combined with nanocarriers to deliver anti-tumor drugs, achieving tumor specific drug delivery [10]. The active targeting moieties specifically bind to the target molecules on the surface of the drug or its carrier, enabling the drug to be targeted to specific tissues or organs, achieving active targeting. Active targeting strategy can avoid the role of mononuclear phagocytosis system and maximize drug accumulation at the target site, thereby improving the anti-tumor efficacy of drugs [12]. The follicle-stimulating hormone receptor (FSHR), characterized by a G protein-coupled receptor structure comprising seven transmembrane domains, is located on the cell surfaces of reproductive organs such as the ovaries, testes, and other related tissues [13,14]. The follicle-stimulating hormone (FSH) is a heterodimeric glycoprotein composed of non-covalently bound α and β subunits [15]. The α subunit is encoded by a single gene shared by FSH, the luteinizing hormone (LH), the human chorionic gonadotropin (hCG), and the thyroid-stimulating hormone (TSH). The β subunit is specific to FSH. The association of FSH β with the FSHR in the cell membrane affects target cells, such as granulosa cells and Sertoli cells. FSHβ 33–53 and FSHβ 81–95 peptides promote paclitaxel-loaded NP entry into the FSH receptor (FSHR)-positive ovarian tissue to achieve excellent anti-tumor effects [16]. Thus, targeting therapy mediated by FSHR has a high potential for ovarian cancer treatment [13,15].

**Figure 1:**
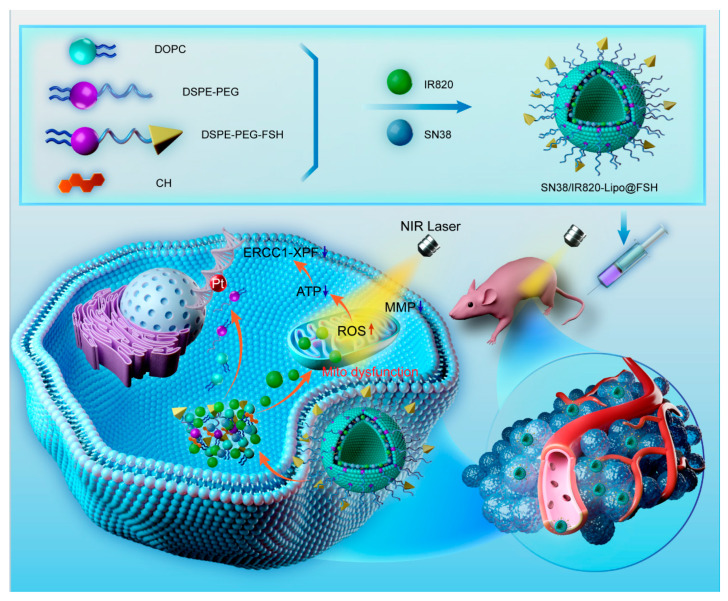
Preparation of SN38/IR820-Lipo@FSH and synergistic effects of chemotherapy and phototherapy in ovarian cancer.

We developed an ovarian cancer-targeting drug delivery system based on the FSHβ (33–53) peptide. The lipophilic chemotherapeutic drug SN38 and the photosensitizer IR820 were loaded into the phospholipid bilayer of the liposomes. IR820 converts NIR into heat and produces ROS. It not only causes damage to intracellular components, leading to cell death, but also enhances the sensitivity of tumors to chemotherapy drugs. These effects induce the release and uptake of chemotherapy drugs and increase sensitivity to chemotherapy drugs in ovarian cancer cells, as illustrated in Figure 1. Chemotherapy and phototherapy act synergistically, and this dual-delivery liposome may be a promising strategy for tumor treatment.

## 2. Materials and Methods

### 2.1. Materials

SN38 was procured from Dalian Meilun Biotechnology Co., Ltd. (Dalian, China). The FSH β (FSH) peptide (YTRDLVYKDPARPKIQKTCTF) was custom-synthesized by Nanjing TGpeptide Biotechnology Co., Ltd. (Nanjing, China). New indocyanine green (IR820) and Coumarin-6 (98%) were obtained from J&K Scientific Co., Ltd. (Beijing, China). DSPE-PEG_2000_, DSPE-PEG_2000_-Mal, 2-dioleoyl-sn-glycero-3-phosphocholine (DOPC), and cholesterol were purchased from AVT (Shanghai, China) Pharmaceutical Technology Co., Ltd. (Shanghai, China). The Cell Counting Kit-8 (CCK-8) was obtained from Dojindo Laboratories (Kumamoto, Japan). The Fluorescein Isothiocyanate (FITC)-Annexin V/Propidium Iodide (PI) apoptosis detection kit was acquired from Beyotime Biotechnology Co., Ltd. (Shanghai, China). The ROS Detection Kit was purchased from Enzo Life Sciences Co., Ltd. (Beijing, China). The Calcineurin-AM/PI Staining Kit and 4′,6-diamidino-2-phenylindole (DAPI) were purchased from Solarbio Life Sciences Co., Ltd. (Beijing, China). The 1,1-dioctadecyltetramethyl indotricarbocyanine iodide (DIR) was obtained from Biotium Inc. (Hayward, CA, USA). Chloroform and methanol (HPLC grade) were purchased from Sigma-Aldrich Co. (St Louis, MO, USA). Fetal bovine serum (FBS) was purchased from GIBCO LLC. (Grand Island, NY, USA). RPMI 1640 medium, DMEM medium, and phosphate-buffered saline (PBS) were obtained from Thermo Fisher Scientific Co., Ltd. (Beijing, China).

### 2.2. Cells and Animals

High FSHR-expressing mouse A2780 cells and low FSHR-expressing HepG2 and C26 cells were purchased from the Department of Pathology at Peking Union Medical College Institute of Medicinal Biotechnology. A2780 cells and C26 cells were cultured in RPMI-1640 medium with 10% FBS at 37 °C and 5% CO_2_. HepG2 cells were cultured in DMEM media with 10% FBS at 37 °C and 5% CO_2_ in a humidified atmosphere. All experiments were performed in cells at the logarithmic growth stage.

Female BALB/c Nude mice (initial weight 18–20 g) were sourced from Vital River Laboratory Animal Technology Co., Ltd. (Beijing, China) and were cared for under suitable conditions. All animal experiments were carried out in accordance with the guidelines established and approved by the Laboratory Animal Ethics Committee of the Institute of Materia Medica in the Chinese Academy of Medical Sciences and Peking Union Medical College.

### 2.3. DSPE-PEG_2000_-FSH Synthesis and Characterization

The synthesis of DSPE-PEG_2000_-FSH was based on the method by Hong et al. [16] with slight modifications. The FSH-Cys cysteine residue was coupled to DSPE-PEG_2000_-MAL to make DSPE-PEG_2000_-FSH. FSH-Cys and DSPE-PEG_2000_-MAL (molar ratio 1:1) were dissolved in a HEPES buffer at pH 8.0, and nitrogen was stirred at room temperature for 48 h. The reaction was conducted in dialysis bags in water. The unreacted FSH peptide was removed by aeration in water through a dialysis bag (MWCO: 2500) for 48 h. The final product was obtained by freeze-drying. The synthesis of DSPE-PEG_2000_-FSH was validated through ^1^H-NMR (400 MHz, Varian Medical Systems, Inc., Palo Alto, CA, USA) and matrix-assisted laser desorption/ionization time-of-flight mass spectrometry (MALDI-TOF-MS) (4800 Plus, Applied Biosystems Inc., Waltham, MA, USA).

### 2.4. Liposome Preparation with or without FSH Peptide Modification

SN38/IR820-Lipo@FSH liposomes were prepared by thin-film hydration method. After dissolving DOPC, cholesterol, DSPE-PEG_2000_, DSPE-PEG_2000_-FSH, SN38, and IR820 in chloroform, the organic solvents were removed by rotary evaporation at 37 °C. The lipid membranes were hydrated with deionized water under spinning for 30 min. The whole mixture was sonicated at 65 W for 10 min with 2 s of sonication and 2 s of a break in an ice bath using an ultrasonic cell pulverizer (Scientz 950E; Ningbo Scientz Biotechnology Co., Ltd., Ningbo, China). The unbound SN38 and IR820 were removed by filtration through a 0.22 μm nitrocellulose membrane. Samples were stored at 4 °C. Blank-Lipo, SN38-Lipo@FSH, IR820-Lipo@FSH, and SN38/IR820-Lipo (without FSH peptide) were prepared using the same method.

### 2.5. Characterization of the Liposomes

The average particle size, polydispersity index, and zeta potential of SN38/IR820-Lipo@FSH were measured using dynamic light scattering (DLS) and electrophoretic light scattering (Zetasizer Nano ZS90, Malvern Instruments, Malvern, UK). The structure and morphology were examined using transmission electron microscopy (JEM-1400PLUS, JEOL Ltd., Tokyo, Japan). The SN38 and IR820 concentrations were determined using high-performance liquid chromatography (HPLC, Agilent 1200 infinity; Agilent Technologies, Santa Clara, CA, USA) and UV-VIS spectrophotometry (TU-1810, Pulse Analyzer General Instrument Ltd., Beijing, China), respectively. The detection wavelength of SN38 was 265 nm, and methanol:water (70:30) was used as the mobile phase. Free SN38 and IR820 were separated from the liposomes using minicolumn centrifugation [17]. The entrapment efficiency (EE%) was calculated as follows:

EE (%) = (weight of encapsulated drug)/(weight of total drug) × 100%

To determine the stability of SN38/IR820-Lipo@FSH, the prepared solution was stored at 4 °C for 7 days in PBS or PBS containing 50% of serum. The color, transparency, particle size, zeta potential, and PDI of SN38/IR820-Lipo@FSH were recorded during the days.

The in vitro release of drugs from SN38/IR820-Lipo@FSH in a simulated physiological environment was evaluated using dialysis. SN38/IR820-Lipo@FSH solution (0.5 mL) was placed in a dialysis bag (MW: 10 kDa; MYM Biological Technology Co., Ltd., Hyderabad, India), and the dialysis bag was immersed in 10 mL of PBS (pH 7.4) containing 0.5% (*v*/*v*) Tween 80. The dialysis solution was stirred at 100 rpm for 72 h at 37 °C, and 0.5 mL aliquots were removed at 0.5, 1, 2, 4, 6, 8, 10, 12, 24, 48, and 72 h. The aliquot volume was replaced with fresh medium. The SN38 and IR820 concentrations in the samples were determined using HPLC and UV-VIS spectrophotometry, respectively. The cumulative release of SN38 and IR820 over time was calculated.

### 2.6. Interaction of SN38/IR820-Lipo@FSH with FSHR In Vitro

Since ovarian cancer cells are highly expressed with FSHR, we investigated the interaction between the FSH target peptide in SN38/IR820-Lipo@FSH and the FSHR on the surface of A2780 ovarian cancer cells. SN38/IR820-Lipo and SN38/IR820-Lipo@FSH were introduced to A2780 cells and incubated at 37 °C for 24 h to assess their binding to the FSHR. Following incubation, cells were rinsed with PBS and lysed using lysis buffer. The cell lysates were incubated for 30 min at 4 °C and then centrifuged for 15 min at 12,000× *g*. Subsequently, the protein lysates were separated on sodium dodecyl sulfate-polyacrylamide gels and transferred to polyvinylidene difluoride membranes. The membranes were incubated overnight at 4 °C with AC-TIN (GB12001, Servicebio, Beijing, China) and FSHR antibodies (1:1000; 2808s, Cell Signaling, Shanghai, China). Following washing, the membranes were incubated with goat anti-rat IgG antibody for 1 h, and observed using a chemiluminescence (ImageQuant LAS 4000 mini, Fuji, Japan) [7,9,10,11,18].

### 2.7. Cellular Uptake Analysis

As a drug delivery system, the efficiency of liposome internalization by target cells is crucial [19]. Cou-6, a fluorescent probe and laser dye, was used to track liposome uptake [20]. Cou-6-labeled liposomes with or without FSH modifications (Cou-6-Lipo and Cou-6-Lipo@FSH) were prepared. A2780 cells, HepG2 cells, and C26 cells (1.5 × 10^5^ cells/well) were seeded on circular glass slides in 12-well plates. After incubation at 37 °C for 24 h, the cells were treated with free Cou-6, Cou-6-Lipo, or Cou-6-Lipo@FSH (Cou-6, 5 µg/mL) for 4 h. After washing three times with the cold PBS buffer, the cells were fixed with 4% paraformaldehyde. Cells were stained with DAPI to visualize the cell nuclei. Finally, cells were imaged with a confocal laser scanning microscope (Carl Zeiss LSM 710; Carl Zeiss Microscope, Jena, Germany).

The uptake efficiencies of Free Cou-6, Cou-6-Lipo, and Cou-6-Lipo@FSH in A2780 cells were quantified using a flow cytometer (Becton Dickinson, Franklin Lakes, NJ, USA). A2780 cells were seeded in 12-well plates (1.5 × 10^5^ cells/well). After 24 h, the cells were exposed to free Cou-6, Cou-6-Lipo, or Cou-6-Lipo@FSH (Cou-6, 1 µg/mL) in a serum-free culture medium at 37 °C for 4 h. Subsequently, cells were washed with PBS, and the fluorescence intensity of the cells was measured using flow cytometry.

### 2.8. ROS Generation

To measure ROS production, A2780 cells (1.5 × 10^5^ cells/well) were seeded into 12-well plates and cultured for 24 h at 37 °C and 5% CO_2_. After washing three times with cold PBS, the cells were treated with Blank-Lipo, SN38-Lipo@FSH, IR820-Lipo@FSH, SN38/IR820-Lipo, SN38/IR820-Lipo@FSH (SN38: 1 µg/mL; IR820: 1 µg/mL), or physiological saline (control) for 4 h. The cells were treated with 1 mL of the ROS detection probe DCFH-DA (25 μM) and incubated for 10 min. The photic group was exposed to 5 min of 808 nm laser irradiation (0.5 W/cm^2^). Cells were treated following the kit instructions, and the fluorescence resulting from ROS production was observed using an inverted fluorescence microscope (CKX41, Olympus, Tokyo, Japan).

### 2.9. Cell Apoptosis Assay

Cell apoptosis was qualitatively evaluated using Calcein-AM/PI staining. A2780 cells were seeded into 12-well plates and incubated for 24 h. Cells were treated with Blank-Lipo, SN38-Lipo@FSH, IR820-Lipo@FSH, SN38/IR820-Lipo, or SN38/IR820-Lipo@FSH (SN38 and IR820 both 1 μg/mL) diluted in fresh culture medium. After 4 h of treatment, the cells were irradiated for 3 min (808 nm, 0.5 W/cm^2^). After 24 h, Calcein-AM/PI live/dead cell staining was performed and photographed.

The apoptosis rate of A2780 cells was quantified using an Annexin V-FITC/PI apoptosis detection kit. A2780 cells (1.5 × 10^5^ cells/well) were inoculated in 6-well plates and cultured for 24 h. Cells were treated as previously described, and irradiated for 3 min using an 808 nm laser (0.5 W/cm^2^). After 24 h, the cells were treated with 0.25% EDTA-free trypsin. Annexin V-FITC/PI apoptosis detection was performed according to the manufacturer’s protocol. Cells were immediately analyzed using flow cytometry.

### 2.10. Wound Healing Assay

The migratory capacity of tumor cells following treatment with various liposomes was assessed. A2780 cells were seeded in 12-well plates and cultured until a monolayer was formed. After the scratch, the cells were treated with free Blank-Lipo, SN38-Lipo@FSH, IR820-Lipo@FSH (Laser +), SN38/IR820-Lipo (Laser +), or SN38/IR820-Lipo@FSH (Laser +) (with both SN38 and IR820 at 0.2 μg/mL). Fresh medium was used as a control. After 24 h, migration of cells in the wound was observed using an inverted microscope. (Olympus, Hamburg, Germany).

### 2.11. Cell Viability Assay

The cytotoxic effects of the blank vectors on A2780 cells were measured using the CCK-8 assay. A2780 cells (4 × 10^3^ cells/well) were seeded in a 96-well plates. After 24 h, the cells were treated with 0.001–50 μg/mL Blank-Lipo in fresh media for 24 or 48 h. After replacing the micelle with CCK-8 solution, the cells were incubated for 3 h at 37 °C. The optical density was measured at 450 nm using a Synergy H1 Microplate Reader (BioTek Instruments, Inc., Winooski, VT, USA). Cells treated with culture media only were used as the control. Each group consisted of three parallel samples.

The inhibitory effects of Free SN38, SN38-Lipo@FSH, Free IR820, IR820-Lipo@FSH, SN38/IR820-Lipo and SN38/IR820-Lipo@FSH on A2780 cell proliferation were determined using the CCK-8 assay. A2780 cells (4 × 10^3^ cells/well) were seeded into 96-well plates. After 24 h, cells were treated with Free SN38, SN38-Lipo@FSH, Free IR820, IR820-Lipo@FSH, SN38/IR820-Lipo and SN38/IR820-Lipo@FSH (SN38: 1 µg/mL; IR820: 1 µg/mL), then the photic group was exposed to 5 min of 808 nm laser irradiation (0.5 W/cm^2^). The control group was included in the analysis. Cells were irradiated with an 808 nm laser (0.5 W/cm^2^) for 3 min per well, and the cells were incubated at 37 °C for another 24 or 48 h. CCK-8 reagent was added to each well, and the optical density was measured at 450 nm. Cell viability (%) was calculated as follows:

Cell viability (%) = [(A test − A blank)/(A control − A blank)] × 100%

### 2.12. In Vivo Imaging

BALB/c Nude mice were injected in the right flank with 200 μL of A2780 cells (1 × 10^8^ cells/mL). Mice were randomly divided into three groups (n = 3/group) when the tumor volumes reached 100 mm^3^. Free DIR, DIR-loaded liposomes (DIR-Lipo), or DIR-loaded liposomes with FSH (DIR-Lipo@FSH) were injected intravenously at a dose of 0.1 mg/kg of DIR. Mice were anesthetized and imaged 1, 4, 8, and 24 h after injection (Caliper Life Sciences Inc., Mountain View, CA, USA). Hearts, livers, spleens, lungs, kidneys, and tumor tissues were collected from the mice 24 h after injection, and analyzed using Living Image software (Version 4.3.1; Caliper Life Sciences Inc.).

### 2.13. In Vivo US-Induced ROS Generation

To evaluate US-induced ROS generation at the tumor sites, the tumor-bearing BALB/c nude mice (n = 3/group) were intravenously injected with PBS, Free IR820, SN38/IR820-Lipo, and SN38/IR820-Lipo@FSH (IR820 dose of 10 mg/kg). After 24 h, the mice were anesthetized with chloralic hydras and injected with 50 µg DCFH-DA intra-tumorally. After 10 min, the tumor sites of the mice were irradiated (808 nm, 0.5 W/cm^2^) for 10 min and then, the mice were sacrificed. The collected tumors were flash frozen and observed by CLSM.

### 2.14. Anti-Tumor Efficacy and Safety Assessment

The tumor-bearing mice were randomly divided into six groups (n = 4/group). Blank-Lipo, SN38-Lipo@FSH, IR820-Lipo@FSH, SN38/IR820-Lipo, SN38/IR820-Lipo@FSH (SN38, 10 mg/kg; IR820, 10 mg/kg), or saline (negative control) were injected intravenously. All animals were given the drug via the tail vein every 4 days for a total of 5 doses. Mice in the SN38/IR820-Lipo@FSH group were subjected to 808 nm laser irradiation (0.5 W/cm^2^) for 10 min on the second day after the injection. The irradiation treatment was repeated once every three days for a total of five treatments. Body weights and tumor dimensions (measured with a caliper) were recorded after each treatment.

Three days after the final treatment, mice were euthanized by cervical dislocation after blood collection from the orbital sinus. The main organs were taken for HE staining, and the tumor tissues were taken for HE and TUNEL testing to examine the cellular apoptosis in the tumor tissues. Lung tissues were fixed in Bouin’s solution for 24 h and immersed in 50% ethanol for 2 h to detect tumor lung metastases. Blood samples were collected to measure blood urea nitrogen, creatinine, aspartate transaminase, and alanine transaminase levels.

### 2.15. Statistical Analysis

All data are presented as the means ± standard deviations. GraphPad Prism software 8.0.2 was used for data analysis. Groups were compared using two-tailed Student’s *t*-tests or one-way analysis of variance. Differences between groups were considered statistically significant at * *p* < 0.05, ** *p* < 0.01, and *** *p* < 0.001.

## 3. Results

### 3.1. Synthesis and Characterization of DSPE-PEG_2000_-FSH

**Figure 2:**
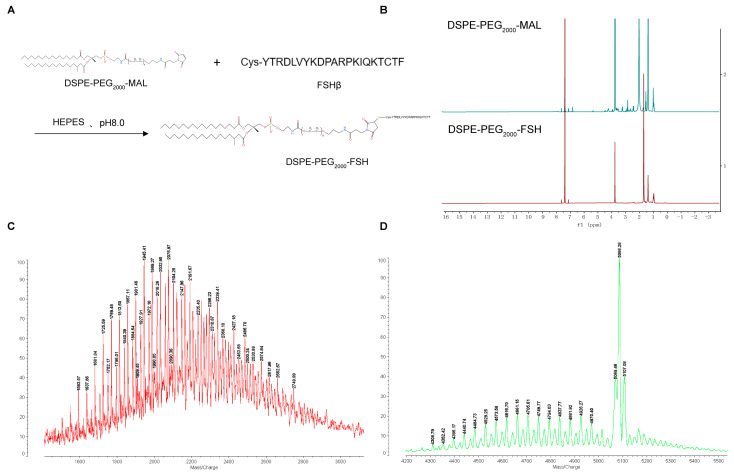
(**A**) Synthesis route of DSPE-PEG_2000_-FSH; (**B**) ^1^H NMR spectra of DSPE-PEG_2000_-Mal and DSPE-PEG_2000_-FSH; (**C**,**D**) MALDI-TOF-MS spectra of DSPE-PEG_2000_-FSH.

The synthesis of DSPE-PEG_2000_-FSH is depicted in Figure 2A. The FSH peptide was coupled with DSPE-PEG_2000_-MAL through an addition reaction. The maleimide group on DSPE-PEG_2000_-MAL reacted with the thiol group on the FSH peptide with nitrogen protection, resulting in the formation of DSPE-PEG_2000_-FSH. The ^1^H-NMR spectrum of DSPE-PEG_2000_-MAL and DSPE-PEG_2000_-FSH are shown in Figure 2B. The maleimide double bond was observed at 6.74 ppm in DSPE-PEG_2000_-MAL but not in DSPE-PEG_2000_-FSH, confirming the successful conjugation. The MALDI-TOF-MS spectrum showed that DSPE-PEG_2000_-MAL had an average relative molecular weight of about 2000~2200 Da, and the synthesized DSPE-PEG_2000_-FSH had an average relative molecular weight of about 4600~4700 Da. Considering that the molecular weight of FSH is 2543 Da, the increased molecular weight coincides with the molecular weight of FSH, indicating that FSH has successfully connected to DSPE-PEG_2000_ (Figure 2C,D). 

### 3.2. Characterization of Liposomes

**Figure 3:**
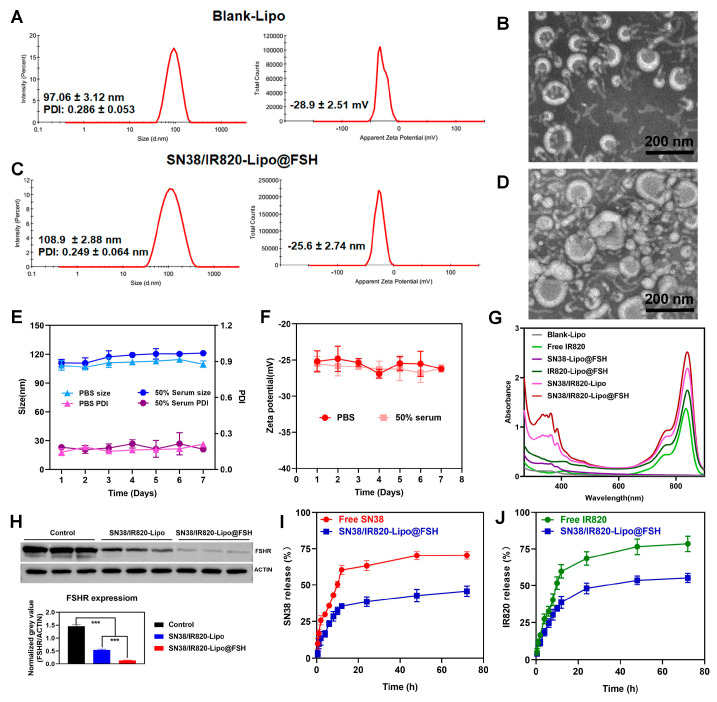
Characterization of Blank-Lipo and SN38/IR820-Lipo@FSH. (**A**,**B**) The average size and zeta of Blank-Lipo and TEM images of Blank-Lipo (scale bar: 100 μm); (**C**,**D**) The average size and zeta of SN38/IR820-Lipo@FSH and TEM images of SN38/IR820-Lipo@FSH (scale bar: 100 μm); (**E**,**F**) Stability of SN38/IR820-Lipo@FSH in 4 °C within 7 days; (**G**) UV/VIS absorption spectra; (**H**) Western blotting analysis of FSHR content, *** *p* < 0.001; (**I**,**J**) The drug release behavior of SN38/IR820-Lipo@FSH within 72 h.

DLS revealed that the Blank-Lipo had an average particle size of 97.06 ± 3.12 nm and a PDI of 0.286 ± 0.053 (Figure 3A). SN38/IR820-Lipo@FSH, encapsulating SN38 and IR820, formed stable nanoparticles with an average particle size of 108.9 ± 2.88 nm and an average PDI of 0.249 ± 0.064 (Figure 3C). The zeta potentials of Blank-Lipo and SN38/IR820-Lipo@FSH were −28.9 ± 2.51 mV and −25.6 ± 2.74 mV, respectively. The particle size distribution was relatively narrow, which may reduce non-specific organ uptake and enhance accumulation at the tumor site. Compared to liposomes with a positively charged surface, liposomes with a negatively charged surface can improve their stability in circulation. Positively charged liposomes may interact with anionic components in the blood, such as plasma proteins, and are absorbed and cleared by the mononuclear phagocytic cell system (MPS). SN38/IR820-Lipo@FSH morphology was also examined using transmission electron microscopy. As shown in Figure 3B,D, the images revealed that both the Blank-Lipo and SN38/IR820-Lipo@FSH exhibited a smooth, spherical structure with uniform dispersion, consistent with the results obtained from DLS analysis. In addition, we investigated the stability of liposomes in PBS and serum-containing PBS. We observed the color change and precipitation of liposome solution, and measured the particle size and potential. The results showed that SN38/IR820-Lipo@FSH had no color change and precipitation formation during the observation period. As shown in Figure 3E,F, minimal changes in the size and zeta potential were detected after storing the liposomes for 7 days at room temperature in PBS or PBS containing 50% serum. The EE percentages for SN38 and IR820 in SN38/IR820-Lipo@FSH were 90.2 and 91.5%, respectively. Moreover, the successful encapsulation of IR820 was confirmed by the characteristic UV-VIS absorption peaks at 820 nm (Figure 3G).

The FSHR content in vitro was confirmed and analyzed by Western blotting analysis. Control, SN38/IR820-Lipo and SN38/IR820-Lipo@FSH had FSHR (71~78 kDa) protein bands (Figure 3H). However, the normalized gray value of FSHR absorbed on the SN38/IR820-Lipo@FSH was lower than the FSHR absorbed on the SN38/IR820-Lipo. These results suggest that SN38/IR820-Lipo@FSH binds to the FSHR receptor due to the FSH peptide modification, decreasing the FSH peptide concentration and increasing the specific capture of FSHR receptor-targeted apolipoproteins.

In order to investigate the release behavior after drug loading, the release behavior of SN38 and IR820 from SN38/IR820-Lipo@FSH was investigated at 37 °C in PBS (pH 7.4, 0.5% Tween 80). As shown in Figure 3I,J, the cumulative release of SN38 and IR820 was less than 20%, indicating excellent in vivo sustained release functionality of the liposomes.

### 3.3. Cellular Uptake Studies

**Figure 4:**
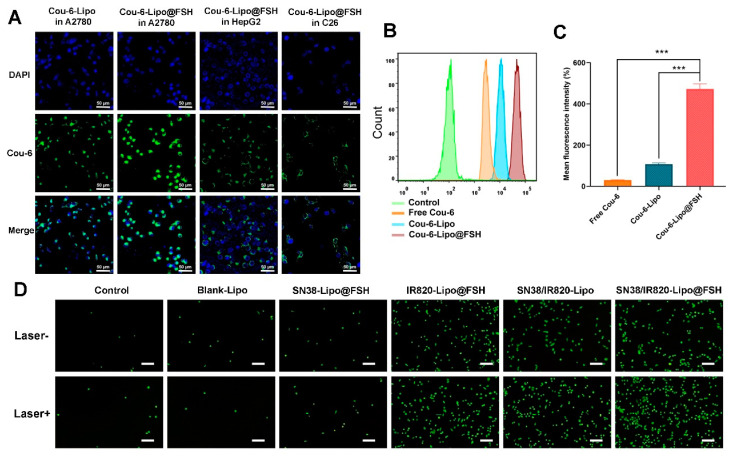
Cellular uptake of SN38/IR820-Lipo@FSH. (**A**) Cell uptake of the liposomes in A2780, HepG2 and C26 cells using CLSM (Scale bar: 50 μm); (**B**) Quantitative analysis of cellular uptake using flow cytometry; (**C**) Mean fluorescence intensity. (**D**) Inverted fluorescence microscope image of ROS (Scale bar: 200 μm). Data are shown as mean ± SD (n = 3). *** *p* < 0.001.

Cou-6-Lipo and Cou-6-Lipo@FSH were prepared to track liposome uptake in A2780, HepG2, and C26 cells. The uptake and efficiency of SN38/IR820-Lipo@FSH were determined using flow cytometry and confocal microscopy. As shown in Figure 4A, the green fluorescence signal of Cou-6 increased in A2780 cells after incubation with Cou-6-Lipo@FSH for 4 h. In contrast, the fluorescent signal increased only slightly in HepG2 and C26 cells after treatment with Cou-6-Lipo@FSH. Furthermore, the uptake of liposomes with the FSH peptide was significantly higher than the uptake of liposomes without the FSH peptide in A2780 cells. These results indicate that the FSH peptide can accurately target the FSHR receptor in A2780 cells to enhance specificity. The results obtained from flow cytometry were consistent with the confocal microscopy results. As shown in Figure 4B,C, the uptake of Cou-6-Lipo@FSH was 4.04 times higher than the uptake of Cou-6-Lipo in A2780 cells. These results indicate that liposomes with the FSH peptide enhance uptake, selectivity, and efficiency in tumor cells with high FSHR expression.

### 3.4. ROS Generation

DCFH-DA is a cell-permeable, intracellular reactive oxygen species (ROS) detection probe (Ex/Em = 488/525 nm). ROS generation in A2780 cells in response to near-infrared laser irradiation was detected using this probe and fluorescence microscopy. After incubating A2780 cells with different liposomes for 4 h, ROS production increased significantly in the groups that underwent 808 nm laser irradiation compared with ROS production in the non-irradiated groups (Figure 4D). Furthermore, the SN38/IR820-Lipo@FSH group exhibited higher ROS generation in response to 808 nm laser irradiation than the Control, Blank-Lipo, SN38-Lipo@FSH, IR820-Lipo@FSH and SN38/IR820-Lipo groups. These results show that IR820 PDT activity was activated in response to 808 nm laser irradiation, resulting in the generation of ROS.

### 3.5. In Vitro Cell Apoptosis Study

**Figure 5:**
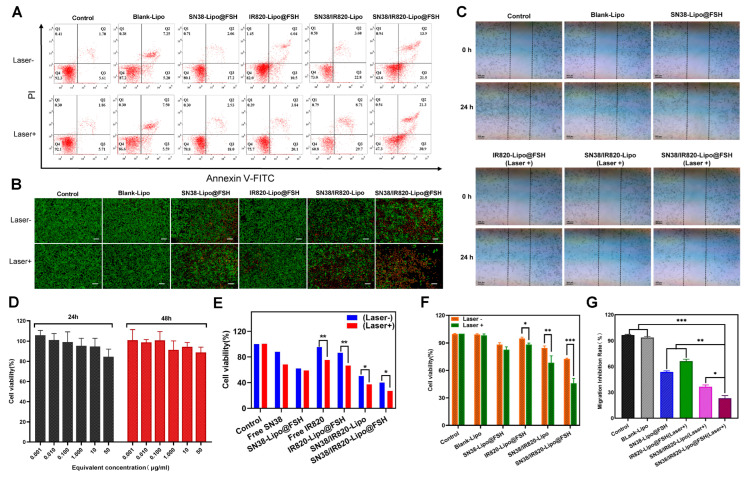
(**A**,**F**) The flow cytometry results of A2780 cell apoptosis and the percentage of early and late apoptosis after the 48 h treatment and near-infrared laser irradiation with Control, Blank-Lipo, SN38-Lipo@FSH, IR820-Lipo@FSH, SN38/IR820-Lipo and SN38/IR820-Lipo@FSH; (**B**) Live/dead detection was performed on A2780 cells treated with different liposomes using the Calcein AM/PI dual staining (Scale bar: 200 μm); (**C**,**G**) Wound healing Assay; (**D**) In vitro cytotoxicity of Blank-Lipo on A2780 cells; (**E**) Inhibitory capacity of different formulations against A2780 cells proliferation. Data are shown as mean ± SD (n = 3). * *p* < 0.05, ** *p* < 0.01, *** *p* < 0.001.

The apoptotic rate of cells was measured using annexin V-FITC/PI double staining and flow cytometry. Apoptotic rates were low in the Control and Blank-Lipo groups. Apoptosis after SN38/IR820-Lipo@FSH treatment was significantly higher than apoptosis after SN38-Lipo@FSH, IR820-Lipo@FSH, or SN38/IR820-Lipo treatments (Figure 5A,F). The apoptosis rate after light exposure in the SN38/IR820-Lipo@FSH treatment group was 52.7%, higher than other groups.

Calcein-AM staining of live cells and PI staining of dead or late apoptotic cells were visualized with an inverted fluorescence microscope. As shown in Figure 5B, SN38-Lipo@FSH, SN38/IR820-Lipo, and SN38/IR820-Lipo@FSH exhibited weak red signals in the absence of light. After light exposure, SN38/IR820-Lipo@FSH exhibited a significant red fluorescent signal, indicating significantly lower cell viability compared with the other groups. These results show that the strong cellular uptake induced by FSH peptide targeting induced more apoptosis in the SN38/IR820-Lipo@FSH treatment group.

### 3.6. Wound Healing Assay

To further investigate the anti-metastatic effect of SN38/IR820-Lipo@FSH, a wound-healing assay was employed to assess random cell migration and invasion. As depicted in Figure 5C,G, scratches in the PBS and Blank-Lipo groups exhibited gradual healing. Following treatment with SN38-Lipo@FSH or IR820-Lipo@FSH (Laser +), the scratches remained visible. However, scratches in the SN38/IR820-Lipo (Laser +) or SN38/IR820-Lipo@FSH (Laser +) group were distinctly visible due to the greater cytotoxicity and notable inhibition of A2780 cell migration compared to the other groups. Importantly, SN38/IR820-Lipo@FSH under irradiation demonstrated a more potent anti-metastatic effect than SN38/IR820-Lipo alone. The enhanced anti-metastatic effect of SN38/IR820-Lipo@FSH was attributed to its high cellular internalization in A2780 tumor cells facilitated by FSH-mediated active targeting.

### 3.7. Cell Viability Assay

We assessed the viability of A2780 cells treated with Blank-Lipo using the CCK-8 assay. No significant cytotoxic effects were observed in A2780 cells after treatment with Blank-Lipo. Cell survival rates exceeded 90% after 24 or 48 h incubation (Figure 5D). Thus, Blank-Lipo is safe and biocompatible for in vivo applications.

The CCK-8 assay was also used to assess the inhibitory effects. As shown in Figure 5E, both light-illuminated groups had a stronger inhibitory effect on cell proliferation than the unilluminated group. SN38/IR820-Lipo and SN38/IR820-Lipo@FSH significantly inhibited the proliferation of A2780 cells compared with the other groups, and SN38/IR820-Lipo@FSH inhibited the cell proliferation more strongly than SN38/IR820-Lipo after 808 nm laser irradiation, with inhibition rates of less than 30%. The high cellular uptake of SN38/IR820-Lipo@FSH enhanced its effect on A2780 cells. IR820-Lipo@FSH’s high cellular uptake enhanced its anti-proliferative effect in A2780 cells, which is consistent with the increased uptake caused by FSH peptide targeting.

### 3.8. In Vivo Biodistribution

**Figure 6:**
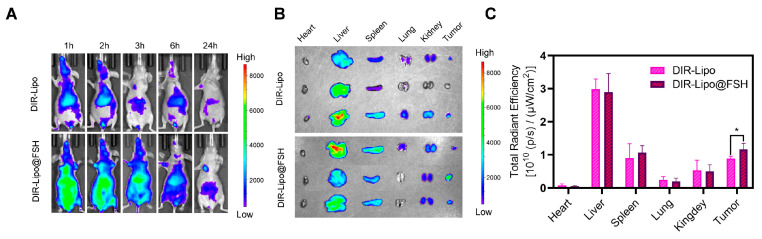
Biodistribution of SN38/IR820-Lipo@FSH in vivo. (**A**) Fluorescence images captured in vivo depict A2780-bearing BALB/c Nude mice treated with DIR-Lipo and DiR-Lipo@FSH. Images were taken at 1, 2, 3, 6, and 24 h after injection; (**B**) Fluorescence images of the excised tumors and major organs were obtained at 24 h after injection; (**C**) Quantitative Region of Interest (ROI) analysis was performed on the excised tumors and major organs at 24 h after injection. Data are shown as mean ± SD (n = 3). * *p* < 0.05.

An in vivo imaging system was employed to determine the biodistribution of DIR-Lipo and DIR-Lipo@FSH in mice bearing A2780 subcutaneous tumors. In vivo images of mice acquired at different time points and corresponding fluorescence quantification results. As shown in Figure 6A, DIR-Lipo was rapidly eliminated after injection. DIR-Lipo@FSH significantly accumulated at the tumor site at various time points following systemic administration; the fluorescence signal lasted 24 h, indicating the crucial role of FSH peptide in tumor targeting. The fluorescence distribution in organs and tumors was determined 24 h after the last liposome administration. The fluorescence intensity was significantly higher in tumors from mice treated with DIR-Lipo@FSH compared with the fluorescence intensity in tumors from mice treated with DIR-Lipo (Figure 6B,C). These findings indicate that DIR-Lipo@FSH exhibits excellent targeting properties. 

### 3.9. In Vivo ROS Generation Efficiency

**Figure 7:**
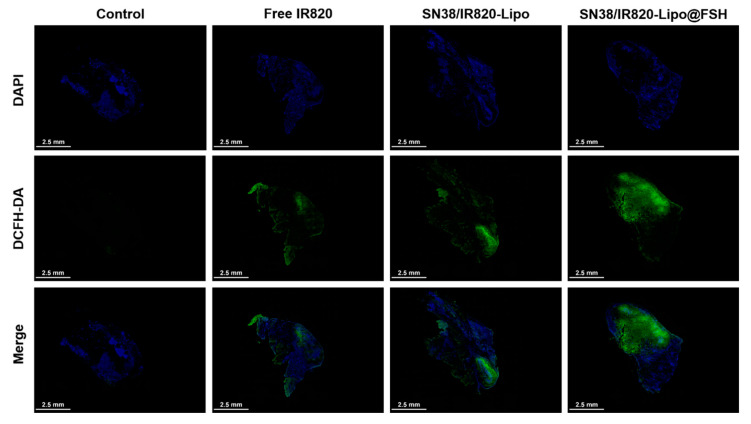
In vivo ROS generation using CLSM (Scale bar: 2.5 mm).

In order to determine the effect of ROS generated by IR820, we observed the levels of DCFH-DA in vivo. As can be seen in Figure 7, strong green fluorescence of DCF was detected throughout the tumor sections of the SN38/IR820-Lipo@FSH group under irradiation, indicating ROS generation in vivo. However, negligible levels of DCF green fluorescence was observed in the PBS group. Compared with the PBS control group, although the DCF fluorescence intensity of the Free IR820 and SN38/IR820-Lipo groups was strong, it was significantly lower than that of the SN38/IR820-Lipo@FSH, indicating that the IR820 photosensitizer in the SN38/IR820-Lipo@FSH was successfully excited under infrared irradiation and produced ROS in vivo.

### 3.10. In Vivo Anti-Tumor Efficacy

**Figure 8:**
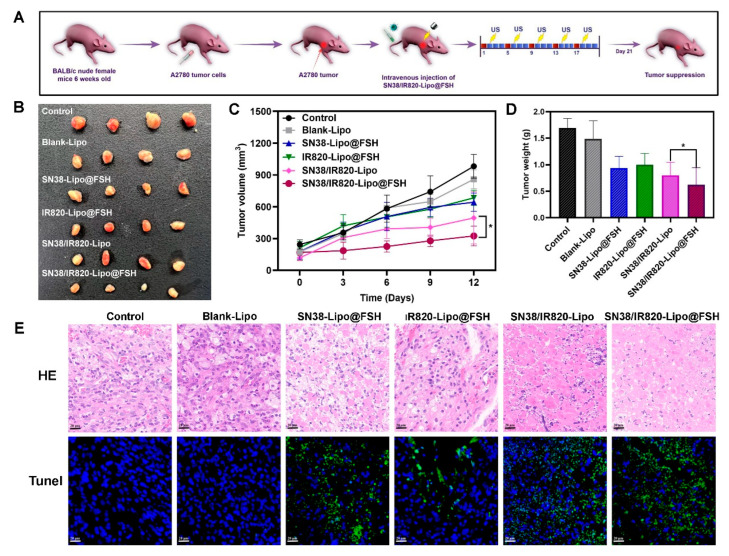
Anti-tumor Evaluation of SN38/IR820-Lipo@FSH in vivo. The A2780-bearing BALB/c Nude mice were treated with Saline, Blank-Lipo, SN38-Lipo@FSH, IR820-Lipo@FSH, SN38/IR820-Lipo and SN38/IR820-Lipo@FSH. (**A**) Experimental procedure; (**B**) Image of isolated tumor tissues of mice; (**C**) Tumor volume of mice; (**D**) Weight of isolated tumors; (**E**) H&E and TUNEL assay of tumor tissues isolated from mice (Scale bar: 50 μm). Data are shown as mean ± SD (n = 4). * *p* < 0.05.

An ovarian cancer subcutaneous tumor mouse model was established to evaluate liposome anti-tumor effects. Mice were separated into six groups (n = 4/group), which were intravenously injected with Blank-Lipo, SN38-Lipo@FSH, IR820-Lipo@FSH, SN38/IR820-Lipo, SN38/IR820-Lipo@FSH, or saline and exposed to 808 nm laser irradiation the following day (As shown in Figure 8A). The mice were euthanized three days after the final injection, and the major organs and tumors were collected. Tumor suppression was enhanced after light exposure in mice treated with FSH peptide-modified liposomes; tumor volumes were lower in the SN38/IR820-Lipo@FSH group compared with tumor volumes in the other groups (Figure 8B–D). Tumor volumes in the SN38-Lipo@FSH and IR820-Lipo@FSH groups were not significantly lower than tumor volumes in the saline group. These results suggested that the SN38/IR820-Lipo@FSH delivery system enhanced the in vivo anti-tumor activity of the drug.

To confirm the effects of SN38/IR820-Lipo@FSH on tumor apoptosis in vivo, tumor tissues were analyzed using TUNEL assays. More TUNEL-positive cells were detected in tumors from mice treated with SN38/IR820-Lipo@FSH than in mice from the other groups (Figure 8E), indicating widespread necrosis and apoptosis in the tumor tissue. This result suggests that SN38/IR820-Lipo@FSH can effectively penetrate the tumor tissue and induce cell death. In contrast, the SN38/IR820-Lipo group showed a lower cell apoptosis rate compared with the SN38/IR820-Lipo@FSH group. Furthermore, tumor localization of SN38-Lipo@FSH and IR820-Lipo@FSH was limited compared with SN38/IR820-Lipo@FSH. Thus, mice treated with SN38/IR820-Lipo@FSH exhibited the best anti-ovarian cancer activity among all the groups.

### 3.11. Safety Assessment

**Figure 9:**
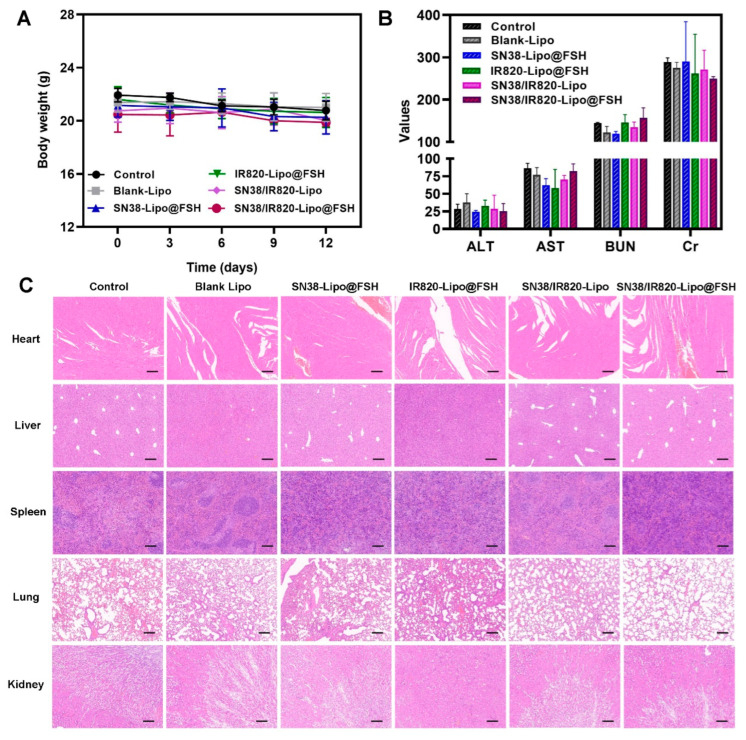
Anti-metastasis evaluation of SN38/IR820-Lipo@FSH in vivo. The A2780-bearing BALB/c Nude mice were treated with Saline, Blank-Lipo, SN38-Lipo@FSH, IR820-Lipo@FSH, SN38/IR820-Lipo and SN38/IR820-Lipo@FSH. (**A**) Body weight of mice; (**B**) ALT, AST, BUN and Cr from the bloodsamples isolated from mice; (**C**) H&E staining of tumor and organs (Scale bar: 200 μm). Data are shown as mean ± SD (n = 4).

Weight loss reflects cancer proliferation and systemic drug toxicity, and preventing weight loss is essential for cancer survival [21]. Changes in mouse body weight after treatment with the various formulations are shown in Figure 9A. Significant changes in body weight did not occur in any treatment group, suggesting that the liposomes reduced the toxicity and side effects of the drugs and were, therefore, safe in mice. Hematological analysis of blood samples from mice treated with the different formulations showed that serum alanine aminotransferase, aspartate aminotransferase, creatinine, and blood urea nitrogen were stable, and all the parameters fell within the safe range for each group (Figure 9B). These findings imply that SN38/IR820-Lipo@FSH may lower SN38 toxicity and improve chemotherapy drug safety, making it a valuable potential delivery strategy.

To assess the ability of SN38/IR820-Lipo@FSH to inhibit metastasis, the major organs were stained with hematoxylin and eosin. No visible inflammatory damage was detected in the hearts, livers, spleens, and kidneys of any group, indicating that the agents were safe and biocompatible (Figure 9C). Moreover, metastatic foci appeared in the lungs of the saline, SN38-Lipo@FSH, IR820-Lipo@FSH, and SN38/IR820-Lipo groups, but no tumor metastases were detected in mice treated with SN38/IR820-Lipo@FSH, suggesting that SN38/IR820-Lipo@FSH inhibited tumor metastasis.

## 4. Discussion

Ovarian cancer is a highly malignant epithelial cell tumor that lacks typical clinical manifestations. Therefore, by the time of diagnosis, the tumor has reached a level that is difficult to treat, bringing enormous life and psychological pressure to patients. In this study, SN38 and IR820 were co-loaded into liposomes and surface modified with FSH peptides with an ovarian cancer active targeting function, resulting in ovarian cancer active targeting. After intravenous injection through the tail vein, the liposome circulates in the blood and selectively targets the site of ovarian cancer tumors, providing more precise therapeutic effects and minimizing side effects on other tissues.

SN38 is an active metabolite of the first-line drug irinotecan, with strong anti-tumor effects. However, due to the limited water solubility, unstable properties, drug resistance, and adverse reactions of SN38, its widespread clinical application is limited [22]. Nano-delivery system can increase the solubility of SN38, reduce its toxic side effects on normal tissues, and improve anti-tumor efficacy. In addition, combining PDT with chemotherapy to improve the sensitivity and anti-tumor efficacy of chemotherapy drugs is a promising application strategy [23]. Therefore, in this study, we simultaneously encapsulated SN38 and IR820 in liposomes to enhance the anti-tumor activity of these two drugs and effectively deliver them to the tumor tissues.

The ability of liposomes to accurately reach the tumor site and successfully release drugs is the key to exert its cytotoxic effect. DSPE-PEG_2000_ is a safe and widely used phospholipid polymer in the field of drug delivery, with biocompatibility, biodegradability, and amphiphilicity [24]. DSPE-PEG_2000_-FSH fabricated liposomes can target ovarian cancer cells overexpressed with FSHR receptors. This strategy increases cellular uptake of the drugs, reduces damage to normal cells, and achieves more effective and precise treatment. The experimental results showed that compared with non-targeted liposome, FSH-modified liposome exhibited enhanced cellular uptake and reduced systemic side effects. After PDT irradiation, IR820 produces ROS, enhancing the sensitivity of tumors to chemotherapy drugs, thereby promoting tumor cell apoptosis and improving anti-tumor efficacy. Both in vitro and in vivo studies have shown that SN38/IR820-Lipo@FSH significantly inhibits the growth of A2780 ovarian cancer cells. H&E and TUNEL experimental results of tumor tissue showed that SN38/IR820-Lipo@FSH group efficiently induced cell apoptosis and necrosis. Importantly, there is no significant damage to other organs, indicating SN38/IR820-Lipo@FSH is expected to become a safe and effective new type of anti-ovarian cancer treatment.

## 5. Conclusions

In this study, we developed SN38/IR820-Lipo@FSH, which combined PDT and chemotherapy to treat cancer. FSH-modified liposomes were used to target ovarian cancer and tumor cell targeting was demonstrated in vitro and in vivo. SN38 and IR820 were efficiently encapsulated within the liposomes, thereby enhancing the water solubility and stability of SN38 in aqueous solutions and significantly reducing toxicity in mice. Moreover, SN38/IR820-Lipo@FSH generated ROS after laser irradiation, leading to cell death and enhanced anticancer effects. In vitro studies demonstrated that SN38/IR820-Lipo@FSH effectively promoted drug uptake by tumor cells, leading to decreased proliferation and increased apoptosis of ovarian cancer cells. Crucially, in vivo experiments demonstrated that SN38/IR820-Lipo@FSH effectively delivered targeted SN38 and IR820 to the tumor site, leading to the inhibition of tumor growth and metastasis. Furthermore, the novel carrier was simple to prepare, and encapsulation efficiency was high. This novel drug delivery system combined PDT and other chemotherapeutic drugs to treat ovarian cancer and demonstrated clinical potential.

## Data Availability

All data available are reported in the article.
